# Proteomics Approaches to Ecoimmunology: New Insights into Wildlife Immunity and Disease

**DOI:** 10.1093/icb/icaf044

**Published:** 2025-05-19

**Authors:** Amanda Vicente-Santos, Natalia Sandoval-Herrera, Gábor Á Czirják, Benjamin A Neely, Daniel J Becker

**Affiliations:** School of Biological Sciences, University of Oklahoma, Norman, OK 73019, USA; Department of Wildlife, Fish, and Environmental Studies, Swedish University of Agricultural Sciences, Umeå 907 36, Sweden; Department of Wildlife Diseases, Leibniz Institute for Zoo and Wildlife Research, Berlin 10315, Germany; Chemical Sciences Division, National Institute of Standards and Technology, Charleston, SC 29412, USA; School of Biological Sciences, University of Oklahoma, Norman, OK 73019, USA

## Abstract

Understanding wildlife immune responses is crucial for assessing disease risks, environmental stress effects, and conservation challenges. Traditional ecoimmunology approaches rely on targeted assays, which, while informative, often provide a fragmented and species-limited view of immune function. Proteomics offers a powerful alternative by enabling the high-throughput, system-wide quantification of immune-related proteins, providing a functional perspective on immunity that overcomes many limitations of conventional methods. However, proteomics remains underutilized in ecoimmunology despite its potential to enhance biomarker discovery, host–pathogen interaction studies, and environmental health assessments. This perspective highlights proteomics as a transformative tool for ecoimmunology, disease ecology, and conservation biology. We discuss its unique advantages over other -omics approaches, including its ability to capture realized immune function rather than inferred gene expression, its applicability to diverse wildlife taxa, and its potential for longitudinal immune monitoring of individuals using minimally invasive sampling. We also address key challenges, including limited genomic reference resources, sample constraints, reproducibility issues, and the need for standardized protocols. To overcome these barriers, we propose practical solutions, such as leveraging proteomes of closely related species for annotation and using their annotated genomes as search spaces for peptide mapping. Additionally, we highlight the importance of alternative quality control strategies and improved data-sharing practices to enhance the utility of proteomics in wildlife research. To fully integrate proteomics into ecoimmunology, we recommend expanding public reference databases for non-model species, refining field-adapted workflows, and fostering interdisciplinary collaboration between ecologists, immunologists, and bioinformaticians. By embracing these advancements, the field can leverage proteomics to bridge the gap between molecular mechanisms and ecological processes, ultimately improving our ability to monitor wildlife health, predict disease risks, and inform conservation strategies in the face of environmental change.

## Introduction

Ecoimmunology studies how immune function interacts with ecological and evolutionary processes, particularly in natural environments and under environmental stressors (Brock et al. 2014). It has emerged as a fundamental field in understanding how wildlife adapts to environmental changes, particularly those driven or exacerbated by human pressures, such as pollution and climate change (Brock et al. 2014; Ohmer et al. 2021). By studying immune function in free-living animals, ecoimmunologists can identify early warning signs of disease risks and population decline, enabling proactive conservation and management strategies (Downs et al. 2014; Ohmer et al. 2021). However, wildlife research presents distinct and inherent challenges, especially when prioritizing the implementation of non-lethal, minimally invasive, and field-friendly methods. Wildlife researchers often obtain fragmented, context-limited data, largely due to the inherent constraints of fieldwork. Critical variables such as individual health history, prior pathogen exposures, and physiological condition remain difficult to assess, all of which limit our ability to fully capture immune function in an ecological context (Martin et al. 2006; Blackwell and Garcia 2022). Therefore, wildlife researchers aim to gather as much information as possible within the limited time frame and resources we have for observation.

Traditionally, ecoimmunology has relied on conventional assays such as leukocyte counts, acute phase protein quantification, and bacterial killing assays—methods that, while valuable, provide a narrow view into immune function rather than a system-wide perspective (Boughton et al. 2011). Other widely used approaches, such as *in vivo* immune challenges and quantifying antibodies or inflammatory responses (Demas et al. 2011), also provide important but still selective insights into vertebrate immunity. Each assay costs time and resources and often draws from limited sample volumes, meaning that researchers can typically only obtain a small number of measurements for any given individual. Fortunately, the advent of more sophisticated techniques, particularly transcriptomics, has helped overcome some of these limitations by providing data on the expression of thousands of immune genes (Zylberberg 2019). On the other hand, proteomics, another -omics technique, has received less attention despite some clear benefits and advantages over complementary transcriptomics (Box 1). Proteomics is another data-rich approach that instead directly measures the abundance of proteins, capturing an organism’s immune landscape in real time (Armengaud 2022). This approach holds immense promise for diverse applications in wildlife research, including but not limited to tracking immune responses to environmental stressors, longitudinal monitoring, elucidating host–pathogen interactions, and identifying biomarkers of disease susceptibility and conservation physiology (Dheilly et al. 2014; Ohmer et al. 2021).

**Box 1: Proteomics vs. transcriptomics in wildlife research**

Transcriptomics and proteomics provide complementary insights into wildlife immune function but differ in what they measure and how they are utilized. Transcriptomics evaluates gene expression by quantifying mRNA levels, offering a snapshot of potential protein synthesis (Hegde et al. 2003). This method is valuable for detecting immune activation and regulatory responses, but it does not consider post-transcriptional modifications, protein degradation, translation efficiency, or protein interactions—factors that affect realized immune function (Hegde et al. 2003; Buccitelli and Selbach 2020). As a result, gene expression and protein abundance tend to show positive but often weak correlations, as many regulatory mechanisms occur post-transcriptionally (Edfors et al. 2016; Liu et al. 2016).

Proteomics, in contrast, directly measures proteins, the functional molecules that drive immune responses (Manzoni et al. 2018). This makes proteomics more reflective of real-time physiological states, capturing immune activity under dynamic environmental conditions. Additionally, blood-based proteomics provides a systemic view of an individual's physiology, as the blood proteome includes not only proteins produced from blood cells but also secreted proteins from organs such as the liver (Uhlén et al. 2019). However, proteomics requires well-annotated reference databases for accurate peptide identification, which can be challenging in non-model wildlife species (Kumar et al. 2016).

Current proteomic methods can provide near-complete coverage of the proteome (Sinitcyn et al. 2023), although the dynamic range of the proteome in biofluids such as blood typically results in the identification of far fewer proteins than gene transcripts from RNA-Seq (Anderson and Anderson 2002; Uhlén et al. 2019). This discrepancy arises because biofluid proteomes primarily capture secreted proteins, whereas transcriptomes reflect gene expression from cells present in the fluid, such as blood cells in whole blood. Additionally, while transcriptomics is generally more cost-effective and benefits from well-developed pipelines (Armengaud 2022), proteomics provides a closer link to functional immunity and host-pathogen interactions. Integrating both approaches can provide a holistic view of wildlife ecoimmunology, bridging the gap between immune potential and realized immune responses (Zylberberg 2019).

Both approaches differ in their sample requirements, which is particularly important in studies prioritizing non-lethal sampling (Neely et al. 2021). Transcriptomics typically requires high-quality RNA, which degrades quickly and can be difficult to extract from non-invasive samples like feces, urine, or feathers (Kumar et al. 2016). For blood, relatively large blood volumes may be needed for sufficient RNA extraction (e.g., 80–200 μL; Huang et al. 2016), which may be limiting for many smaller wildlife. In contrast, proteomic analyses can be performed on a broader range of sample types, including blood, saliva, and even shed skin, making them more adaptable for field studies (Armengaud 2022). Sample volumes can also be smaller than some transcriptomic applications (e.g., 2 μL of serum or plasma; Neely et al. 2021). While transcriptomics often relies on freshly preserved tissues (though it is increasingly applied to blood), common application of proteomics to diverse biofluids, in conjunction with the small sample volumes noted above, can allow researchers to assess immunity repeatedly over time (Neely et al. 2021), reducing the impact on wild populations and improving longitudinal immune monitoring. Additionally, RNA is highly sensitive to degradation from freeze–thaw cycles, making long-term sample storage and repeated analysis more challenging. In contrast, studies on the human proteome suggest protein stability is relatively higher, with limited artifacts introduced by a single freeze–thaw cycle and even minor changes after multiple cycles (Hulmes et al. 2004; Mitchell et al. 2005).

With this Perspective, we aim to highlight proteomics as a transformative tool for ecoimmunology, offering valuable applications to the adjacent fields of disease ecology, ecotoxicology, and conservation biology. By integrating proteomics with traditional immunological assays and other -omics approaches, researchers can build a more holistic framework for understanding immune variation in wild populations. Proteomics has the potential to bridge existing knowledge gaps, foster interdisciplinary collaborations, and advance conservation efforts in an era of rapid environmental change.

### Applications of proteomics to ecoimmunology and conservation

**Fig. 1:**
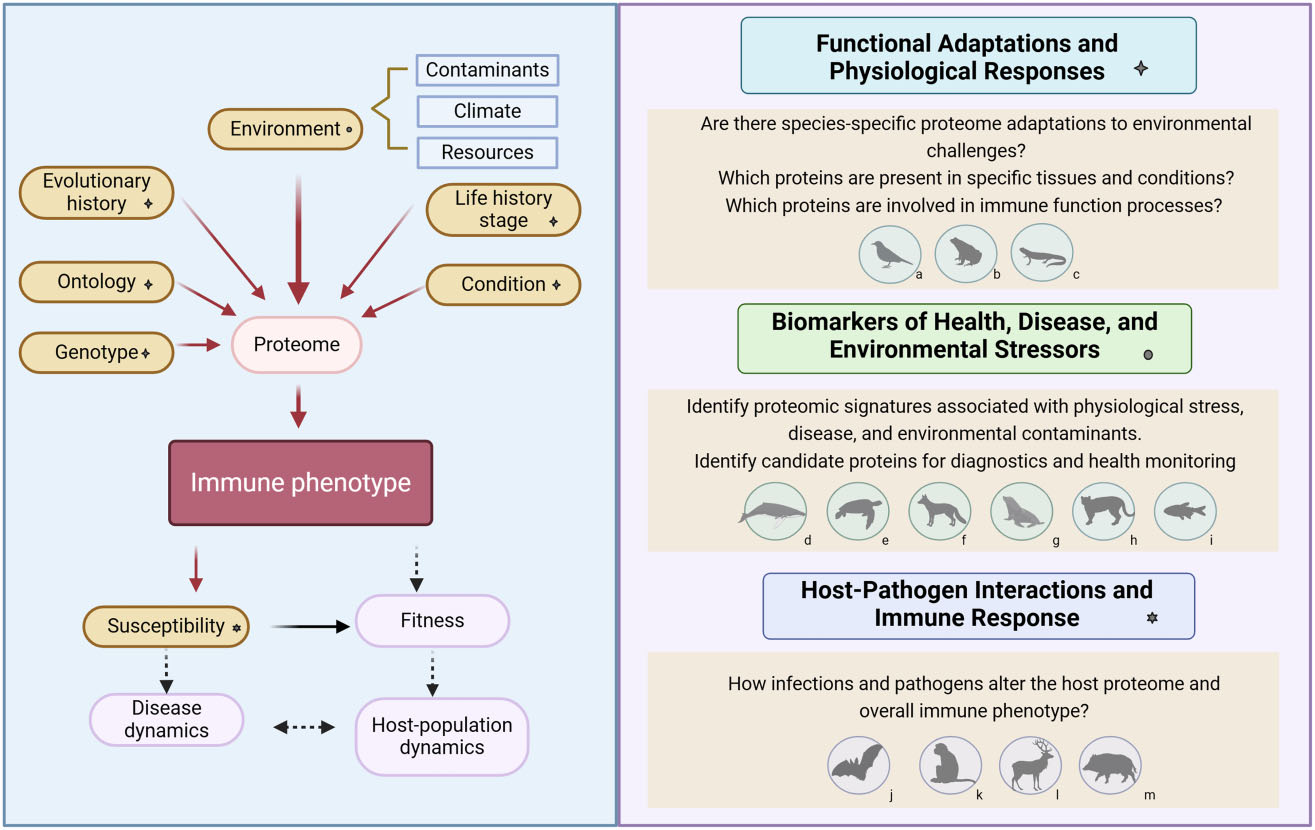
Applications of proteomics in wildlife immunology. The left panel shows the main drivers of intra- and interspecific variance in immune phenotypes and their influence on larger-scale processes (adapted from Schoenle et al. 2018). Yellow boxes indicate drivers that can be studied using proteomics. The right panel shows three main categories of application of proteomics in ecoimmunology and example questions that have been addressed in the literature. The symbols (+, ●, *) match the drivers from the left panel. The animal silhouettes illustrate particular studies as examples of the wide range of species studied, where (a) Otčenášková et al. 2023, (b) Li et al. 2024, (c) Geng et al. 2015, (d) Kershaw et al. 2024, (e: Chaousis et al. 2021, (f) Gillis-Germitsch et al. 2021, (g) Neely et al. 2018, (h) Mangiaterra et al. 2022, (i) Raposo de Magalhães et al. 2020, (j) Vicente-Santos et al. 2023, (k) Ruengket et al. 2023, (l) Kuleš et al. 2024, (m) Kuleš et al. 2021.

Proteomics is a powerful tool for addressing a wide spectrum of questions in ecoimmunology and conservation biology (Fig. 1). Proteomics enables the simultaneous detection and quantification of hundreds to potentially thousands of proteins, providing a broad and less-biased view of the immune system compared to focusing only on a few pre-selected markers and/or specific immune branches. By characterizing the proteome of wildlife species, researchers can explore fundamental aspects of immune function, host–pathogen interactions, and the physiological responses of organisms to environmental stressors. For instance, proteomic analyses of wild bats have revealed an unexpected dominance of guanylate-binding proteins—known antiviral molecules—that are less abundant in human serum, suggesting a previously unappreciated mechanism of viral tolerance in bats (Vicente-Santos et al. 2023). Such insights would have been difficult to obtain using conventional immune assays, which typically target pre-defined proteins. Beyond immune response, proteomics can also be leveraged for pathogen surveillance; prior work on wild vampire bats (*Desmodus rotundus*) mined the serum proteome to detect viral proteins, identifying previously uncharacterized viruses circulating in bat populations (Neely et al. 2021). Proteomics can also facilitate comparative analyses across species, providing insights into evolutionary immunology, resilience to disease, and immune strategies (e.g., Hecht-Höger et al. 2020). Similarly, proteomics can help identify early molecular changes in a host before pathophysiological symptoms appear, thereby improving monitoring for sublethal effects of pollutants, climate change, and other environmental stressors on immune function. For instance, studies on the immune system of fish models have revealed changes in protein abundance that correlate with exposure to pollutants (Henke et al. 2024). Additionally, analyses can identify biomarkers that inform conservation strategies, assess health status in wild populations, and even enhance zoonotic disease prediction, as demonstrated by an illustrative series of case studies in marine mammals (Box 2; Neely et al. 2015, 2018).

**Box 2: Proteomics as a tool for wildlife health and conservation: insights from California sea lions**

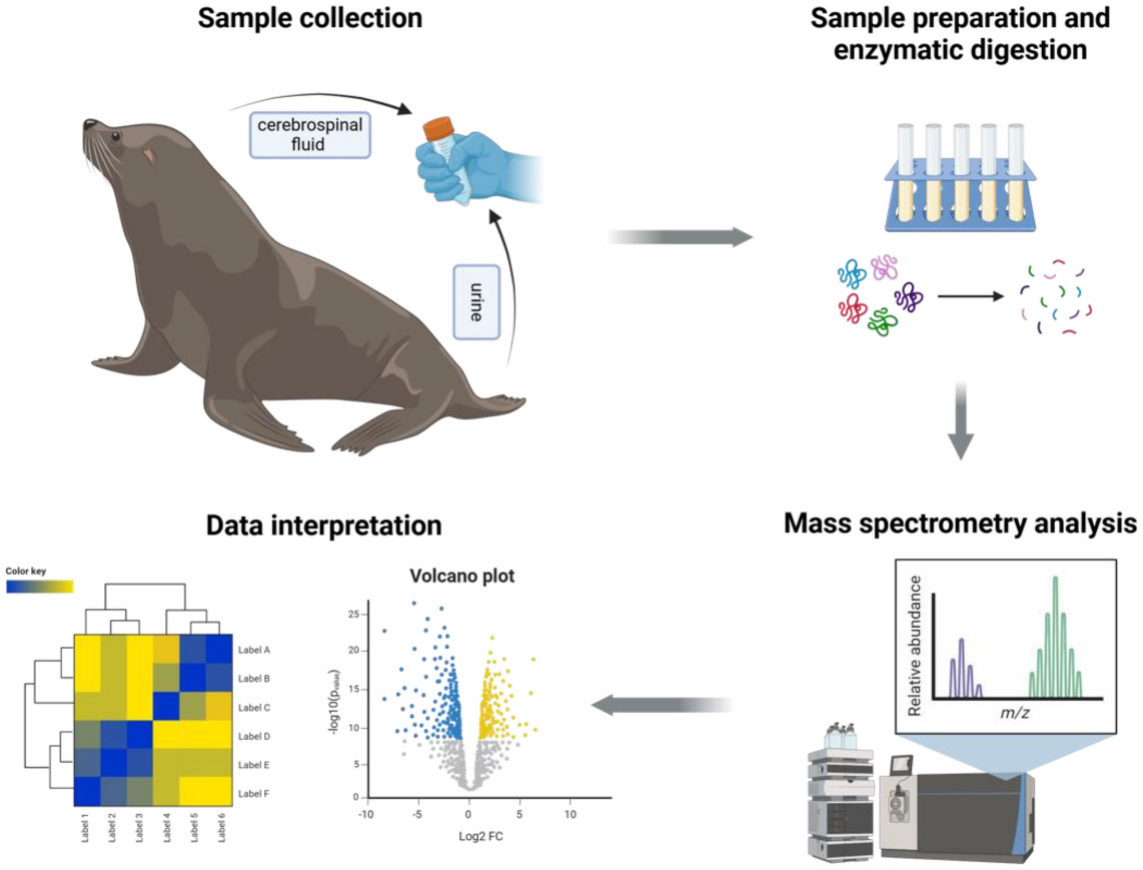

Proteomics has emerged as a powerful tool in wildlife health research, as demonstrated by several studies of California sea lions (*Zalophus californianus*) (Neely et al. 2015, 2018). The 2015 study investigated biomarkers of domoic acid toxicosis using cerebrospinal fluid (CSF) collected aseptically from affected and control sea lions. This invasive but highly targeted approach provided a direct assessment of brain-specific protein alterations associated with neurotoxicity. In contrast, the 2018 study focused on kidney disease linked to *Leptospira* infection, using urine samples from stranded sea lions diagnosed via PCR. Urine, a non-invasive and biomarker-rich biofluid, enabled insights into renal physiology and disease progression. Both studies followed robust sample preparation workflows, including protein precipitation, enzymatic digestion, and mass spectrometry–based proteomic analysis, highlighting the adaptability of proteomics across different sampling strategies.

The results from these studies revealed distinct disease-associated protein signatures. The 2015 study identified 206 CSF proteins, with markers such as gelsolin and reelin linked to domoic acid-induced neurotoxicity. Complement proteins such as C3 and neuronal cell adhesion molecules (NRCAM) further underscored neuroinflammatory and neuronal injury processes. Meanwhile, the 2018 study identified 2,694 urinary protein groups, with 316 displaying significant differences between infected and control sea lions. Notable biomarkers included neutrophil gelatinase-associated lipocalin (NGAL) and osteopontin, which were elevated in leptospirosis cases, while neprilysin, a renin-angiotensin system enzyme, was reduced—mirroring findings in rodent models of kidney disease. Given the absence of a fully sequenced sea lion genome at the time, both studies successfully leveraged composite mammalian protein databases, demonstrating the feasibility of cross-species proteomic comparisons in non-model wildlife species.

These studies illustrate how proteomics can enhance wildlife disease diagnostics and conservation monitoring. The application of CSF proteomics in diagnosing neurodegenerative diseases (Neely et al. 2015) and the validation of urine proteomics as a non-invasive diagnostic tool for renal disease (Neely et al. 2018) demonstrate the broad translational potential of this approach. By adapting similar pipelines to other wildlife diseases and environmental stressors, researchers can develop valuable tools for conservation biology, veterinary medicine, and ecological health assessments. The ability to detect and monitor disease at the molecular level can bridge critical knowledge gaps in wildlife health management, ultimately strengthening conservation efforts.

A major benefit of -omics approaches, including proteomics and transcriptomics, in ecoimmunology is their applicability to various ecological systems. In contrast to many immunological tests that need species-specific validation and reagents, -omics techniques enable high-throughput, comparative analyses across taxa, even in non-model organisms (Heck and Neely 2020). For example, ELISAs are widely used to quantify specific immune proteins, but the lack of species-specific reagents often limits their application in wildlife (Demas et al. 2011). Proteomics thus offers a leapfrog alternative, bypassing the need to develop ELISAs for every species or target protein. This versatility allows for investigating numerous organisms without requiring the costly and time-intensive development of species-specific tools, which can cause significant research delays. However, proteomics holds a unique advantage over transcriptomics in that it directly measures functional proteins, capturing immune responses as they occur rather than inferring them from gene expression data (Box 1). Furthermore, employing tandem mass spectrometry and data-independent acquisition techniques has improved our capability to analyze the proteomes of various species (Henke et al. 2024). Comparison of full proteomes across species could allow the identification of conserved, gained, or lost proteins that may contribute to species-specific immune traits and phenotypic differences in immune responses. These cross-species comparisons can also be enhanced using methods such as protein ranking or endogenous common internal retention time standards (CiRT) for mammals, birds, and reptiles, providing standardized analysis capabilities even for non-model species (Messner et al. 2023).

A significant hurdle in wildlife research is acquiring adequate sample volumes, especially from small or rare species where invasive methods are either undesirable or impractical (Heck and Neely 2020). Proteomics is particularly effective for handling limited sample volumes (e.g., 2 μL of serum or plasma; Neely et al. 2021), making it suitable for non-lethal, minimally invasive collection (Dupree et al. 2020). Analyzing biofluids such as blood, saliva, urine, and even feces minimizes the impact on individual animals and facilitates longitudinal studies that monitor immune responses in the same individual over time (Kennedy 2002). Additionally, sample preservation techniques can be refined for fieldwork in remote or resource-constrained environments (Jiang et al. 2024). Recent innovations in stabilizing protein samples with matrix-based collection systems (e.g., protein saver cards and Mitra microsampling tubes) have removed the need for cold chain storage, thus alleviating logistical challenges and lowering the costs tied to sample transport (Chambers et al. 2013; Lehmann et al. 2017; Skogvold et al. 2023; Thangavelu et al. 2023). Moreover, proteomic applications appear relatively less sensitive to freeze–thaw cycles compared to RNA-based and traditional ecoimmunology methods. Plasma proteomes can remain stable even after multiple freeze–thaw cycles, with limited impact observed after a single cycle (Hulmes et al. 2004) and only minor changes detected after two cycles (Mitchell et al. 2005). Additionally, some protein profiles have been maintained for over four years in long-term storage conditions, making proteomics a particularly resilient approach for field-collected wildlife samples (Mitchell et al. 2005).

Overall, proteomics is a cost-effective and flexible approach that maximizes data acquisition while requiring minimally invasive sampling, small sample volumes, and fewer experimental animals—features especially valuable for vulnerable and understudied species. While proteomic studies can validate existing hypotheses about the molecular mechanisms underlying immunological processes, their exploratory nature enables the discovery of novel insights and formulation of new hypotheses, guiding follow-up research. For instance, in vampire bat sera, proteomic analyses revealed high rank abundance of guanylate-binding proteins (GBPs), a finding that contrasts with their relatively low abundance in the human serum proteome. Given the known antiviral functions of GBPs, this suggests a potential role in bat virus tolerance, providing a new avenue for investigating innate immune adaptations in reservoir hosts (Neely et al. 2021). By applying proteomic techniques to field-based and experimental samples, researchers can gain deeper mechanistic insights into disease ecology, stress and reproductive physiology, and immune adaptation in natural populations. Identifying proteomic biomarkers provides a valuable means of assessing wildlife health, informing conservation strategies, and improving predictions of disease outbreaks, which could potentially impact ecosystems and human health.

### Challenges and workarounds

Given its focus on the proteins involved in immune response, proteomics offers a direct view into the functional state of an organism's immune system and defense capacity; however, its application in wildlife studies faces several significant barriers (Table 1; Katsarou et al. 2021). One major challenge is the limitation of reference databases (Henke et al. 2024). Although the number of annotated genomes continues to grow—reaching 1286 eukaryotic species in NCBI RefSeq by the end of 2024 (Eukaryotic Genome Annotation at NCBI 2025)—most wildlife species still lack the genomic and transcriptomic resources needed for accurate protein identification, unlike well-studied model organisms (Dupree et al. 2020). Consequently, peptides cannot be reliably mapped without the proper search spaces, which limits the interpretability and downstream use of proteomic datasets (Johnson et al. 2020). To overcome this challenge, collaboration within the ecoimmunology community, and adjacent taxonomic fields, is essential. A crucial step forward is expanding species-specific genomic databases, leveraging phylogenetically related species, and integrating *de novo* protein sequencing approaches to fill annotation gaps (Dheilly et al. 2014). Additionally, increasing RNA sequencing efforts, as argued previously (Heck and Neely 2020), will improve existing genomes and facilitate better annotations, complementing ongoing initiatives to generate new references (Larsen et al. 2014; Teeling et al. 2018; Bravo et al. 2021). Strengthening interdisciplinary collaboration and investing in bioinformatics infrastructure will be essential to overcoming these barriers and unlocking the full potential of proteomics in ecoimmunology. However, the absence of an annotated genome for any given wildlife species should not deter researchers from utilizing proteomics, as meaningful results can still be obtained by mapping peptides to well-annotated genomes of closely related species (Heck and Neely 2020). Comparative approaches have successfully identified conserved immune proteins and pathways across taxa (e.g., Otčenášková et al. 2023), demonstrating that cross-species analyses remain a viable and informative strategy.

**Table 1:** Summary of key barriers to implementing proteomics in wildlife studies and proposed workarounds.

**Barrier**	**Description**	**Proposed solution**
Lack of reference genomes	Limited databases hinder peptide identification	Use genomes of closely related species; *de novo* annotation
For non-lethal sampling, reliance on biofluids	Biofluids may not reflect localized immune responses	Combine biofluids with swabs or biopsies; interpret results with caution
Reproducibility	Variation in methods and pipelines	Adopt MIAPE, OORF, HUPO-PSI, QC tools, and standardized protocols
Lack of species-specific antibodies	Limits enrichment and depletion techniques	Use targeted MS (MRM/PRM); develop broad-spectrum or synthetic reagents
Cost and access	Specialized equipment and training required	Foster collaborations and shared core facility access

Another challenge in wildlife proteomics is the reliance on biofluids, such as blood or saliva, rather than specific tissues. While biofluids provide a minimally invasive alternative, they may not fully capture localized immune responses occurring at pathogen entry points (e.g., lungs for respiratory infections, gut for enteric diseases) (Zylberberg 2019). Moreover, immune responses are often compartmentalized, and systemic and local immune responses can be distinct, meaning that protein abundance in circulation does not always reflect tissue-specific immune activation (Heck and Neely 2020). For instance, studies in human immunology have shown that protein composition and abundance can differ substantially between biofluids and tissues, affecting biomarker interpretation and limiting our ability to infer local immune processes from blood-based proteomics alone (Kumar et al. 2016; Buccitelli and Selbach 2020). Future research should, therefore, prioritize comparative analyses across biofluids and tissues to better understand the relationship between systemic and localized immune responses within a single infected organism. Integrating multiple sample types—such as pairing plasma proteomics with non-lethal tissue biopsies or swabs—could improve resolution of findings while maintaining ethical sampling standards (Zylberberg 2019; Heck and Neely 2020).

Another significant hurdle in applying proteomics to wildlife research is reproducibility. Comparing results across studies can be problematic due to variability in experimental design, data processing pipelines, and reporting standards (Poulos et al. 2020; Henke et al. 2024). Proteomic workflows differ in sample preparation, mass spectrometry platforms, and bioinformatics tools, making it challenging to synthesize findings across datasets. This lack of standardization limits the development of universal biomarkers and reduces the utility of proteomics for broader ecological or conservation applications. To address this, adopting standardized reporting frameworks—such as the Minimum Information About a Proteomics Experiment (MIAPE), the OECD Omics Reporting Framework (OORF), and those developed by the HUPO Proteomics Standards Initiative (HUPO-PSI)—can improve transparency and facilitate cross-study comparisons (Taylor et al. 2007; Deutsch et al. 2023; OECD 2023). HUPO-PSI has helped develop widely used data standards, including Sample and Data Relationship Standard (SDRF) for metadata (Dai et al. 2021), mzML for raw mass spectrometry data (Martens et al. 2011), mzIdentML for peptide and protein identification results (Jones et al. 2012), mzQC for QC data (Bielow et al. 2024), and more data standards are in development alongside instrument and acquisition advances. Furthermore, normalization techniques such as those used in assimilating human data across tissues, instruments, and experimental designs (Prakash et al. 2023) can enhance the integration of data from different studies and enable more robust meta-analyses.

Quality control (QC) is another key component of reproducible proteomics, helping to convey confidence in data so that researchers can distinguish biological signals from technical variability and artifacts. Best practices for QC workflows include retention time calibration peptides, whole-proteome digests, and in-run study pool QC samples, all of which are essential for ensuring data consistency (Neely et al. 2024; Tsantilas et al. 2024). Automated QC tracking tools such as AutoQC, QCloud2, and QuiC facilitate early detection of instrumental drift, batch effects, and data anomalies, allowing researchers to troubleshoot issues before they compromise results (Bittremieux et al. 2017; Olivella et al. 2021). Integrating these best practices into wildlife proteomics will be critical as the field expands to larger datasets and multi-laboratory collaborations.

Efforts to improve standardization in wildlife proteomics should also extend to data sharing and protocol transparency. Researchers can contribute by depositing raw and processed proteomics datasets into public repositories, such as MassIVE or ProteomeXchange (Perez-Riverol et al. 2025), facilitating cross-study comparisons and meta-analyses. By providing experimental annotation files such as SDRF (Dai et al. 2021), data can be used and re-analyzed by anyone. Additionally, sharing standardized protocols, methodological details, and data processing pipelines on platforms such as the Open Science Framework (OSF) can improve reproducibility and accelerate methodological advancements in the field.

A further limitation in wildlife proteomics is the lack of species-specific antibodies, which restricts the use of immuno-depletion and enrichment techniques commonly employed in model organisms (Heck and Neely 2020). Without species-specific reagents, detecting low-abundance proteins becomes more challenging, reducing the sensitivity of proteomic analyses and delaying the identification of species-specific biomarkers. Targeted mass spectrometry approaches, such as multiple reaction monitoring (MRM) and parallel reaction monitoring (PRM), offer viable alternatives that bypass the need for such antibodies. These techniques allow for precise quantification of specific proteins and are particularly useful for non-model species, where using broad-spectrum reagents developed for phylogenetically related species, or developing custom antibodies, may not be feasible (Heck and Neely 2020). These assays can be developed to be completely species-specific, high-throughput, and can achieve attomolar detection limits. For instance, past work circumvented the lack of a species-specific adiponectin measurement assay for bottlenose dolphins (*Tursiops truncatus*) by developing a mass spectrometry-based quantification strategy, demonstrating how targeted proteomic workflows can be adapted to study metabolic health in wildlife species (Neely et al. 2013). By integrating such targeted proteomic strategies, researchers can overcome species-specific reagent limitations and improve the detection of functionally relevant proteins in wildlife studies.

However, despite improvements, proteomics remains largely inaccessible to many researchers due to its labor-intensive nature, high costs relative to traditional ecoimmunology assays, and the need for specialized equipment, software, and training. While mass spectrometry technology is becoming more affordable, dedicated proteomics facilities remain concentrated in high-resource institutions, limiting broader access. Furthermore, collaborations with human biomedical proteomics centers—often well-equipped with state-of-the-art mass spectrometry platforms—can be challenging, as these centers may lack the disciplinary background to fully understand the ecological and conservation-driven questions central to wildlife proteomics as well as the particular challenges faced by wildlife samples (e.g., small sample volumes, limited cold chain capacity). Strengthening interdisciplinary collaborations and advocating for the significance of wildlife health research within these facilities will be essential for expanding access to proteomics and unlocking its full potential for ecoimmunology and conservation biology.

## Conclusions and future directions

Proteomics presents a transformative approach to ecoimmunology, offering a high-resolution, systems-level perspective on immune function that overcomes many logistical and interpretive limitations of traditional and individual assays. By enabling cross-species comparisons, capturing functional immune responses, and maximizing data from small sample volumes, proteomics provides a powerful framework for studying host–pathogen interactions, environmental stressors, and conservation-relevant biomarkers in wildlife. Its ability to generate mechanistic insights into disease ecology makes it a valuable tool for both fundamental research and applied medical and conservation efforts.

Realizing the full potential of wildlife proteomics will require stronger interdisciplinary collaboration among ecologists, immunologists, bioinformaticians, and analytical chemists. Integrating proteomics with ecological fieldwork, experimental immunology, and computational modeling will refine our understanding of immune function in natural systems. Continued advancements in mass spectrometry, bioinformatics pipelines, and standardized workflows will further support robust and reproducible applications across diverse taxa (Domon and Aebersold 2006; Jiang et al. 2024). Expanding public databases for non-model species, sharing standardized protocols, and developing scalable workflows tailored to field-collected samples will further facilitate the adoption of proteomics in wildlife research (Heck and Neely 2020; Neely et al. 2023).

For researchers looking to apply proteomics in ecoimmunology, an introduction to key methodologies and best practices is essential. Shuken (2023) and Jiang et al. (2024) provide a comprehensive overview of the fundamental principles of proteomics, detailing methodologies such as shotgun and targeted proteomics, along with their respective advantages in studying complex biological systems. Peters-Clarke et al. (2024) discuss the latest advancements in mass spectrometry techniques that enhance sensitivity and resolution, improving the detection of subtle protein changes in response to environmental stressors. Neely et al. (2024) highlight the importance of quality control measures and reproducibility, which are crucial for ensuring reliable results in ecological and evolutionary studies. Additionally, Dai et al. (2024) demonstrate how it is possible to perform large-scale analysis of multiple datasets and extract meaningful biological insights. Together, these resources offer an essential entry point for researchers seeking to implement proteomics in wildlife studies, providing the necessary conceptual and technical foundation to address pressing ecological and conservation challenges.

As these methodological and analytical improvements progress, proteomics has the potential to reshape ecoimmunology, disease research, and conservation biology. Bridging molecular and ecological scales will enhance our ability to predict wildlife disease risks, monitor population health, and inform conservation strategies in an era of rapid environmental change. Embracing this approach will open new frontiers in understanding how immune systems evolve and function across the tree of life, ultimately advancing fundamental biology, medicine, and practical conservation efforts.

## Authors contributions

A.V.S. and D.J.B. conceived the manuscript, and A.V.S. led its writing and organization. N.S.H., G.Á.C., B.A.N., and D.J.B. contributed to its conceptual development and provided critical revisions. A.V.S. and N.S.H. created the Figs. All authors participated in drafting and editing the manuscript and approved the final version.

## Data Availability

No new data were generated or analysed in support of this research.

## Notes

From the symposium “Applied ecoimmunology in wildlife health and conservation” presented at the annual meeting of the Society for Integrative and Comparative Biology, January 3–7th , 2025.
